# Replication of the GWAS-Identified GALNT13 rs10196189 Polymorphism in Relation to Speed–Power Elite Active Athlete Status and Multidimensional Phenotypic Differences in Chinese Han Males: A Pilot Study

**DOI:** 10.3390/genes16080983

**Published:** 2025-08-20

**Authors:** Lun Chen, Mingrui Wang, Longtianjiao Liu, Xiaoyu Jiang, Zihang Cao, Samuhaer Azhati, Hangyu Chen, Kaixin She, Jinyao Zhu, Ming Chen, Jinda Li, Junhao Kong, Jiahao Zhang, Yuang Yan, Yi Dong, Apudumalike Mieryazi, Songyu Liu, Yanyan Zhang, Yixuan Ma, Lijun Shi

**Affiliations:** 1 Department of Exercise Physiology, Beijing Sport University, Beijing 100084, China; bubbachucks3@hotmail.com (L.C.); wmrbody@bsu.edu.cn (M.W.); 2024210289@bsu.edu.cn (L.L.); chy123@bsu.edu.cn (H.C.); 2022015049@bsu.edu.cn (K.S.); 2022011084@bsu.edu.cn (J.Z.); 2022011875@bsu.edu.cn (M.C.); 2022011872@bsu.edu.cn (J.L.); junhao_kong@outlook.com (J.K.); 2024210310@bsu.edu.cn (J.Z.); 2024210332@bsu.edu.cn (Y.D.); 2024012820@bsu.edu.cn (A.M.); 2024012829@bsu.edu.cn (S.L.); yanyanzhang@bsu.edu.cn (Y.Z.); 2 Top-Tier Performance Physical Fitness Training Center, Beijing 100020, China; 3 Biomechanics Laboratory, Beijing Sports University, Beijing 100084, China; 2024210271@bsu.edu.cn (X.J.); 2024210276@bsu.edu.cn (Z.C.); 2024210272@bsu.edu.cn (Y.Y.); 4 School of Education, Beijing Sport University, Beijing 100084, China; sam@bsu.edu.cn; 5 Laboratory of Sports Stress and Adaptation, General Administration of Sport of China, Beijing Sport University, Beijing 100084, China; 6 Key Laboratory of Physical Fitness and Exercise, Ministry of Education, Beijing Sport University, Beijing 100084, China; 7 Division of Sports Science and Physical Education, Tsinghua University, Beijing 100084, China

**Keywords:** sports genomics, GALNT13, Han Chinese

## Abstract

**Background/Objectives**: Previous multi-ethnic genome-wide association studies (GWAS) have identified the GALNT13 rs10196189 polymorphism as a potential genetic marker linked to sprint–power performance. However, its relevance in East Asian populations, particularly the Han Chinese, remains untested. This study aimed to replicate the association of rs10196189 with elite sprint–power athlete status in Han Chinese males and examine its potential influence on physical performance traits and tissue-specific gene regulation. **Methods**: A total of 188 healthy Han Chinese males (49 elite sprint–power athletes and 139 non-athletic controls) were genotyped using the TaqMan assay. Assessments included strength, sprint, jump, anaerobic power, DXA-derived body composition, and muscle ultrasound. Logistic regression and ROC analyses evaluated the predictive value of rs10196189. Linear regression models adjusted for age and BMI tested genotype–phenotype associations. Tissue expression and functional networks were analyzed using GTEx and HumanBase databases. **Results**: The G allele frequency was significantly higher in athletes (12.2%) than in controls (5.4%, *p* = 0.042). Dominant and additive models effectively predicted athlete status (OR = 2.53–2.58, *p* < 0.05). Although most traits showed no significant associations post-correction, medial gastrocnemius thickness showed a nominal association (β = 0.371, *p* = 0.011). Functional analyses revealed high GALNT13 expression in brain tissue and co-expression networks enriched in synaptic signaling and glycosylation pathways. **Conclusions**: This is the first study to validate the association of GALNT13 rs10196189 with elite athletic status in Han Chinese males. Findings provide novel population-specific evidence and propose tissue-specific glycosylation and neural mechanisms as pathways linking this variant to sprint–power phenotypes.

## 1. Introduction

The development of elite athletic performance is a multifactorial process, shaped by the interaction between environmental influences and an individual’s biological foundation [1]. Although “deliberate practice” has long been considered a key driver of athletic expertise, meta-analyses suggest that it accounts for only about 1% of the variance in performance at the elite level [2]. This finding highlights the importance of integrating genetic, psychological, and physiological factors in talent identification and athlete development frameworks [1,2]. In recent years, sports genomics research has identified over 250 genetic polymorphisms associated with athletic phenotypes, covering traits such as endurance, strength, and power [3].

Single nucleotide polymorphisms (SNPs) are the most common type of genetic variation in the human genome and serve as core markers for phenotype association studies. To date, more than 9 million SNPs have been documented [4]. In the context of athletic performance, increasing evidence suggests that the GALNT13 rs10196189 polymorphism may be an important variant associated with sprint and power traits [5,6,7]. GALNT13 (polypeptide N-acetylgalactosaminyltransferase 13) encodes an enzyme involved in mucin-type O-linked glycosylation. It catalyzes the transfer of N-acetyl-α-D-galactosamine (GalNAc) from UDP-GalNAc to serine (Ser) or threonine (Thr) residues on polypeptide chains, representing the initial step in O-glycan modification [8]. This enzyme belongs to the family of UDP-N-acetyl-α-D-galactosamine:polypeptide N-acetylgalactosaminyltransferases (pp-GalNAc-Ts), of which 20 members have been identified in humans. Among them, GALNT13 (also referred to as pp-GalNAc-T13) is highly specifically expressed in the nervous system, particularly in neuroblastoma cells and primary cultured neurons, whereas its expression is extremely low or undetectable in glioblastoma cells and astrocytes [9]. GALNT13 exhibits pronounced substrate specificity and is capable of catalyzing the formation of multiple consecutive GalNAc-Ser/Thr structures on neural proteins such as syndecan-3. It plays a critical regulatory role in the synthesis of Tn antigens in neurons. In mouse knockout models, the loss of GALNT13 leads to a marked reduction in Tn antigen expression in the cerebellum, suggesting its central role in maintaining glycosylation homeostasis in the nervous system. Enzymologically, GALNT13 is classified under EC 2.4.1.41 (https://www.brenda-enzymes.org/enzyme.php?ecno=2.4.1.41accessed on 6 August 2025), which defines the activity of polypeptide N-acetylgalactosaminyltransferases. Its typical reactions involve the transfer of GalNAc to Ser (Rhea:23956) or Thr (Rhea:52424) residues [10]. The chemical entities involved in these reactions can be further annotated using the ChEBI database: the glycosyl donor UDP-GalNAc is registered as CHEBI:15378, and the glycosylated polypeptide products include several variants such as CHEBI:58223, 67138, and 29999 [11]. The integration of these database identifiers provides a standardized biochemical annotation framework to support future functional predictions and mechanistic studies of GALNT13.

A large-scale cross-ancestry GWAS conducted in 2025 involving elite sprint and power athletes of Jamaican, African-American, and Japanese descent was the first to report a significantly higher frequency of the rs10196189 G allele in elite sprinters compared to geographically matched controls, with genome-wide significance (*p* = 2.13 × 10^−9^) achieved through combined analysis across the three ancestral populations [5]. More recent studies have also found the G allele to be associated with better sprint and countermovement jump (CMJ) performance in British academy football players [6]. In light of these findings, increasing attention has been paid to other members of the N-acetylgalactosaminyltransferase (GALNT) gene family, which share a conserved role in initiating mucin-type O-glycosylation. Notably, a polymorphism in the GALNTL6 gene (rs558129) has been associated with power-oriented performance. The T allele was found to be significantly enriched in elite strength and speed-strength athletes from Spain and Russia and was linked to 5–7% higher anaerobic power values compared to CC homozygotes, suggesting a favorable role in anaerobic capacity [12]. These data collectively support the hypothesis that glycosylation-regulating genes such as GALNT13 and GALNTL6 may contribute to interindividual variability in power–speed traits via modulation of muscle energetics and neuromuscular function. Beyond their athletic relevance, members of the GALNT gene family—including GALNT3, GALNT8, GALNT12, and GALNT13—have also been implicated in inherited disorders such as familial tumoral calcinosis and hyperostosis–hyperphosphatemia syndrome. In silico analyses have predicted that several missense mutations in these genes significantly impair enzymatic function and structural stability, thereby contributing to abnormal phosphate metabolism and tumor-like calcification phenotypes [13]. These pathological insights further underscore the functional importance of O-glycosylation dynamics in both health and disease and provide critical biological context for interpreting genetic associations in athletic populations.

These findings suggest that this variant may serve as a potential genetic marker for explosive athletic phenotypes. However, sports genetics research currently faces two major challenges: insufficient replication rates and population bias. A systematic reproducibility review published in *Sports Medicine* revealed that among 25 replicated studies, only 28% met all three criteria for robust replication—consistency in effect direction, effect size compatibility, and statistical significance [14]. As such, independent replication and external validation of candidate SNPs in diverse populations have become critical imperatives in the advancement of sports genomics.

To date, most research on GALNT13 rs10196189 has been conducted in African and European populations. East Asian populations—particularly the Han Chinese, the world’s largest ethnic group—remain severely underrepresented in both athletic genetic research and reference databases [3,15,16]. Given the population-specific differences in allele frequencies and genetic architecture, replication studies within specific ethnic groups are essential for scientific validity and translational applications. Although population-level genomic resources such as PGG.Han and Han100K have provided valuable data for Chinese genetic research [15,16], no systematic studies have yet examined the distribution and functional relevance of GALNT13 rs10196189 in Chinese athletic populations.

Therefore, the present study aims to assess the predictive and discriminative power of this SNP for identifying elite sprint–power athlete status among Han Chinese males and to explore its potential associations with specific physical performance traits. In addition, by integrating cross-tissue expression and functional data, we sought to elucidate its tissue-specific regulatory mechanisms and contribute key population-level data to the global body of knowledge in sports genomics.

## 2. Materials and Methods

### 2.1. Experimental Approach to the Problem

This study aimed to investigate whether the GALNT13 (rs10196189) polymorphism—previously implicated in neuromuscular traits—is associated with elite sprint–power athlete status and specific phenotypic characteristics in Han Chinese males. Genotypic analyses were conducted in a group of elite sprint–power athletes and compared to an untrained control group to assess differences in physical performance, physiological traits, genotype, and allele frequencies. The objective was to evaluate whether this genetic variant could discriminate athlete status.

**Figure 1:**
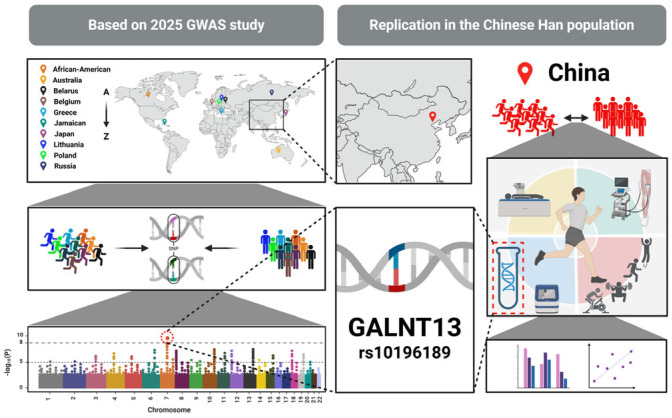
Study framework diagram. The left panel is based on the multi-ethnic GWAS conducted by Wang et al. in 2025, which identified a genetic signal linking GALNT13 rs10196189 to sprint–power performance [5]. The right panel illustrates the replication workflow of this locus in the Han Chinese population, including cohort recruitment, SNP genotyping, phenotypic assessments, and statistical analyses. The aim is to evaluate the applicability and phenotypic relevance of this variant in East Asian populations.

Logistic regression and receiver operating characteristic (ROC) curve analyses were used to assess the predictive utility of rs10196189. Based on the genetic model(s) that significantly predicted athlete status, linear regression models—adjusted for age and BMI—were then applied within the athlete group to assess associations between rs10196189 and physical performance, body composition, and muscle morphological indicators. In parallel, gene function prediction and regulatory mechanism analyses were conducted using tissue-specific expression data to explore the functional relevance of GALNT13 (Figure 1).

Data were collected retrospectively through standardized testing procedures, all conducted under controlled laboratory conditions. The testing protocol was implemented in the following sequence: (1) DXA assessment of body composition and bone mineral density at Beijing Sport University; (2) ultrasound measurement of muscle thickness at the China Institute of Sport & Health Science; (3) standardized warm-up; (4) jump performance tests (CMJ, SJ, and DJ); (5) 20 m sprint; (6) isometric mid-thigh pull (IMTP) test; (7) one-repetition maximum (1RM) squat test; and (8) Wattbike anaerobic power test. All tests were conducted in a climate-controlled indoor setting by professionally trained technicians to ensure procedural consistency and data reliability.

### 2.2. Subjects

A total of 188 healthy Han Chinese males were recruited for this study, including 49 current national-level elite athletes (specializing in sprinting, jumping, or throwing events) and 139 untrained controls (university-aged males with no history of systematic training). All participants were between 18 and 30 years old, with no history of musculoskeletal injuries or chronic diseases at the time of testing (demographic characteristics of participants are presented in Table 1). Prior to participation, written informed consent was obtained from all subjects. The study protocol was reviewed and approved by the Sports Science Experiment Ethics Committee of Beijing Sport University (Approval No. 2025009H), and all procedures were conducted in accordance with the ethical principles of the Declaration of Helsinki.

### 2.3. Procedures

#### 2.3.1. Genotyping

On the day of testing, all participants provided saliva samples using a disposable collection kit (Product No. 180220, Tongshu Gene Technology Co., Ltd., Changzhou, China). Participants were instructed to refrain from eating, drinking, or chewing gum for at least 30 min prior to collection. Each subject self-collected approximately 3 mL of saliva via a funnel-capped collection tube, which automatically mixed with 1 mL of preservation buffer preloaded in the tube. Samples were then sealed and stored at −20°C until DNA extraction. Genomic DNA was extracted using the standard phenol–chloroform method, and DNA concentration and purity were evaluated via NanoDrop™ One UV–Vis spectrophotometer (Thermo Fisher Scientific, Waltham, MA, USA) to ensure suitability for downstream genotyping. This study genotyped one single nucleotide polymorphism (SNP): GALNT13 rs10196189 (A/G). SNP genotyping was conducted using the TaqMan^®^ SNP Genotyping Assay (Assay ID: C_32930554_10, Applied Biosystems, Foster City, CA, USA) on a QuantStudio™ 5 real-time PCR system (Thermo Fisher Scientific, Waltham, MA, USA). For quality control, approximately 10% of samples were randomly selected for repeat genotyping, yielding a 100% concordance rate. Additionally, the Hardy–Weinberg equilibrium (HWE) was tested for the SNP in the non-athlete control group to confirm the validity of genotype distribution and population representativeness [17].

#### 2.3.2. Body Composition and Bone Mineral Density

Dual-energy X-ray absorptiometry (DXA) was used to assess whole-body body composition and bone mineral density. Measurements were performed using the Lunar iDXA scanner (General Electric Healthcare©, Madison, WI, USA). All participants underwent testing in a fasted state in the early morning, with the bladder emptied and all metallic objects removed prior to the scan to minimize measurement artifacts. During scanning, participants lay supine on a flat scanning table, remaining still throughout the procedure. The entire process was conducted by the same professionally trained DXA technician to ensure consistency and data reproducibility. An automated analysis software processed the data in real time based on standardized anatomical regions. Key extracted indicators included DXA body weight (kg); body fat percentage (%); fat mass (g); muscle mass (g); lean body mass (g); body mass index (BMI, kg/m^2^); and bone mineral density (BMD, g/cm^2^). All DXA scans were performed by trained personnel using built-in analysis tools to ensure accurate segmentation and regional quantification, thereby guaranteeing data precision and reliability.

#### 2.3.3. Musculoskeletal Ultrasound Assessment

Musculoskeletal ultrasound assessments were conducted using the Logiqe diagnostic ultrasound system (GE Healthcare, Chicago, IL, USA), equipped with a 7.5 MHz high-frequency linear array probe, offering high-resolution imaging and excellent tissue penetration—particularly suitable for evaluating superficial muscle structures. All scans were performed in a quiet, temperature-controlled indoor environment with participants in a fully relaxed state, assuming standardized postures to ensure anatomical consistency and measurement reproducibility. The evaluations employed B-mode imaging, with both transverse and longitudinal views obtained by an experienced operator. A medical-grade coupling gel was used to facilitate smooth probe movement and eliminate imaging artifacts. The primary measurement was muscle thickness (cm), defined as the vertical distance between the superficial and deep fascia. Target muscles included rectus femoris; vastus medialis; vastus lateralis; semitendinosus; biceps femoris (long head); and gastrocnemius (medial head). Each muscle was measured twice, and if the discrepancy between the two readings exceeded 5%, a third measurement was taken. The average value was used for statistical analysis. All procedures adhered to the guidelines outlined in “*Musculoskeletal ultrasound: technical guidelines*” [18] and were conducted by the same certified examiner to ensure standardized, reliable, and consistent assessments.

#### 2.3.4. Jump Performance

Jump performance was assessed using a KWYP-FP6035-A2 one-dimensional force platform system (Kunwei Yancheng Technology Co., Ltd., Shanghai, China), which supports both wireless and wired dual-channel signal acquisition with a sampling frequency of 1000 Hz. The system offers high sensitivity, making it suitable for capturing rapid force changes during lower-limb explosive movements. Participants performed three standardized jump tests in sequence: Countermovement Jump (CMJ): Starting from a natural standing position with hands on the hips, participants performed a rapid downward movement followed by an immediate upward jump, reflecting eccentric–concentric muscle coordination; Squat Jump (SJ): Participants maintained approximately 90° knee flexion for 2–3 s before jumping, eliminating pre-stretch energy to evaluate pure concentric power; Drop Jump (DJ): From a 30 cm box height, participants dropped naturally onto the platform and performed an immediate rebound jump, assessing reactive strength and explosive power integration during short ground contact times. Each jump test was repeated 2–3 times with 2–3 min of rest between attempts to ensure full recovery. The best performance was recorded for statistical analysis. All tests were supervised by a senior assessor holding NSCA-CPSS (Certified Performance & Sport Scientist) and NSCA-CSCS (Certified Strength and Conditioning Specialist) credentials, ensuring standardized procedures, repeatability, and scientific rigor.

#### 2.3.5. Sprint Performance

Sprint performance was evaluated using a 20 m straight-line sprint test, with timing recorded by a SmartSpeed Pro laser timing system (Fusion Sport, Brisbane, Queensland, Australia). Participants started 1 m behind the start line in a two-point stance and initiated the sprint voluntarily without external cues. Timing gates were placed at 0 m and 20 m, and the system automatically recorded sprint time (s) and calculated mean velocity (m/s). Each participant completed 2–3 sprint trials with 3 min intervals for adequate recovery, and the fastest time was used for statistical analysis.

#### 2.3.6. Maximal Isometric Strength

The isometric mid-thigh pull (IMTP) test was conducted using a dual-platform force measurement system (Model: KWFP6035-A2, Kunwei Yancheng Technology Co., Ltd., Shanghai, China). This system supports both wireless and wired dual-channel data acquisition at a sampling frequency of 1000 Hz, offering high sensitivity for capturing both static and explosive force outputs. Each platform is equipped with an independent sensor that has been factory-calibrated with first-order (A_1_) and second-order (A_2_) coefficients provided by the manufacturer.

During the test, participants placed their feet on the two force platforms and grasped a standardized barbell positioned to simulate the mid-thigh pull stance (with a hip–torso angle of ~120–130° and knee angle of ~125–145°), ensuring a straight back and depressed shoulders. The force sensors recorded output in mV/V, which was converted to actual load (kg) using the calibration formula:

(1) y = A_1_·x + A_2_·x^2^

where x is the sensor signal output and y is the load. The calculated force (kg) was then multiplied by gravitational acceleration (9.81 m/s^2^) to obtain the absolute peak force (N), and further divided by body mass (kg), to yield the relative peak force (N/kg).

Each participant performed two trials, with a 2 min rest between attempts. The maximum value was used for analysis. The test was supervised by the same assessor certified by the NSCA-CSCS (Certified Strength and Conditioning Specialist) to ensure standardized execution, data consistency, and reliability.

#### 2.3.7. Lower-Limb Maximal Strength

The one-repetition maximum (1RM) back squat test was conducted following the standardized protocol recommended by the National Strength and Conditioning Association (NSCA) [19]. Participants performed progressively heavier warm-up sets prior to testing. Rest intervals of 3–5 min were provided between attempts. The maximum load successfully lifted with proper technique was recorded as the final 1RM result.

#### 2.3.8. Tissue-Specific Functional Prediction and Regulatory Mechanism Analysis

To further annotate the functional relevance of GALNT13 and explore its potential biological mechanisms in relation to genotype–phenotype associations, we utilized the Genotype-Tissue Expression (GTEx) database to assess the tissue-specific expression levels of GALNT13 across the human body. The GTEx project is a comprehensive public resource encompassing 44 tissue types, providing RNA-sequencing data from healthy individuals and enabling the analysis of genetic variation effects on gene expression across different tissues [20]. By systematically retrieving GALNT13 expression profiles, we identified tissues with high expression levels, which provided spatial context and biological insight for subsequent functional predictions.

Building upon this expression landscape, we employed Humanbase (https://humanbase.io accessed on 11 July 2025), a platform that integrates machine learning algorithms, including Naïve Bayes, deep learning, and support vector machines, to perform tissue-specific functional predictions for GALNT13. This platform aggregates 987 multi-omics datasets derived from approximately 14,000 scientific publications, covering over 38,000 biological conditions and enabling the construction of tissue-specific functional networks across 144 tissue and cell lineages.

Using the GIANT (Genome-scale Integrated Analysis of gene Networks in Tissues) module within Humanbase [21], we applied the “Functionally Related Genes” tool to identify the top 15 functionally related genes to GALNT13, forming a predicted functional interaction network. Subsequently, Gene Ontology (GO) biological process-based enrichment analysis was conducted on this network using Humanbase’s “Enriched Function” output [22,23]. The goal was to uncover closely associated gene clusters or functional modules relevant to specific biological processes and to identify potentially novel roles of GALNT13 in pathways not yet fully characterized.

In addition, to enhance the biological interpretability of our analysis, we manually integrated two glycosylation-related molecules that are closely associated with the function of GALNT13: B4GALNT1 and MAN2A2. B4GALNT1 (Beta-1,4-N-acetylgalactosaminyltransferase 1) is a key glycosyltransferase involved in N-glycan processing and is closely linked to the pathogenesis of hereditary spastic paraplegia type 26 (HSP26) [24]. A potential functional interaction between this protein and GALNT13 has been predicted by the STRING database [25]. On the other hand, MAN2A2 (alpha-mannosidase II) is a crucial enzyme located in the Golgi apparatus that participates in the trimming steps during N-glycan maturation, and its physical interaction with GALNT13 has been confirmed by experimental data curated in the BioGRID database [26]. Notably, dysfunction of MAN2A2 has been associated with autism spectrum disorder, cognitive impairment, and congenital disorders of glycosylation (CDG) [27,28]. By combining these two molecules with the GALNT13 brain- and skeletal muscle-specific interaction networks constructed by HumanBase, we established an integrated functional network consisting of 30 nodes and conducted biological process enrichment analysis on this gene set, aiming to provide candidate pathways and a network framework for the functional prediction of GALNT13 in exercise-related tissues and for subsequent mechanistic investigations.

### 2.4. Statistical Analysis

All statistical analyses were conducted using SPSS Statistics 27.0 (IBM Corp., Armonk, NY, USA) and R version 4.2.3 (R Core Team, Vienna, Austria) to ensure the accuracy and reproducibility of results. For genetic distribution testing, the genotype distribution of GALNT13 rs10196189 in the control group was assessed using the Hardy–Weinberg equilibrium (HWE) exact test in order to verify the representativeness of the sample and the reliability of genotyping data [17]. Additionally, the minor allele frequency (MAF) from the Han-Chinese Genome Database (PGG.Han v2.0, https://www.biosino.org/pgghan2 accessed on 11 July 2025) [6] was referenced to confirm the population representativeness of the observed allele frequencies, which was cross-validated with the HWE results to strengthen the genetic interpretation and comparability [17]. HWE testing was not applied to the athlete group, as they constitute a selectively recruited non-random population.

To further explore population-level differences in allele distribution, a series of allele frequency comparisons were performed between the Han Chinese control group and multiple global reference populations from the gnomAD database [29], including Total, African-American, Amish, Middle Eastern, South Asian, Remaining (unclassified), Ashkenazi Jewish, European (Non-Finnish and Finnish), Admixed American, and East Asian groups. For each comparison, 2 × 2 contingency tables were constructed based on G and A allele counts, and Pearson’s chi-square tests were conducted to assess statistical differences. The resulting chi-square statistics and *p*-values were extracted to evaluate the significance of inter-population divergence.

In addition, given that the current study included only male participants, a sex-based allele frequency analysis was conducted using gnomAD allele count data from genetically female (XX) and male (XY) individuals. The same chi-square approach was applied to test for potential sex-related differences in allele distribution. Where expected cell frequencies were below 5, Fisher’s exact test was used to ensure the robustness of statistical inference.

Genotype and allele frequency comparisons between speed–power athletes and non-athletic controls were conducted using Pearson’s chi-square test (χ^2^); Fisher’s exact test was applied when expected frequencies were low. All phenotypic indicators (e.g., 1RM squat, isometric strength via IMTP, jump heights, RSImod, 20 m sprint time, and anaerobic power), body composition measures (e.g., DXA-derived body fat percentage, skeletal muscle mass, BMI, and bone density), and muscle morphological parameters (e.g., rectus femoris thickness) were reported as mean ± standard deviation (SD) to indicate central tendency and variability.

Prior to between-group comparisons, Shapiro–Wilk tests were performed to assess normality of each variable within both groups. If both groups satisfied the normality assumption (*p* > 0.05), independent samples *t*-tests were used to compare means; otherwise, the Mann–Whitney U test was applied to avoid bias due to violation of normality assumptions. To assess practical significance, effect sizes were calculated: for normally distributed variables, Cohen’s d was reported (with thresholds |d| ≥ 0.2, 0.5, 0.8, and 1.2 indicating small, medium, large, and very large effects, respectively); for non-normally distributed variables, rank-biserial correlation coefficient (r) was used, with |r| ≥ 0.1, 0.3, and 0.5 corresponding to small, medium, and large effects.

To examine the predictive power of genotypes on athlete status, binary logistic regression models were constructed with athlete status (yes/no) as the dependent variable and genotype as the primary predictor, evaluated under dominant, recessive, and allelic models. Additional models were constructed with age and BMI included as covariates to control for potential confounding effects. Results were presented as adjusted odds ratios (ORs) with 95% confidence intervals (CIs). For models showing statistical significance, receiver operating characteristic (ROC) curve analysis was further conducted, and area under the curve (AUC) values with 95% CIs were reported to evaluate the discriminatory capacity of each model. AUC values were interpreted as follows: 0.5–0.7 (low accuracy), 0.7–0.9 (moderate accuracy), and >0.9 (high accuracy).

For genetic models demonstrating significant effects on athlete classification (e.g., dominant, additive, or recessive models), linear regression analyses were subsequently performed in the athlete group to explore the association between rs10196189 genotypes and performance, body composition, and muscle morphology. Age and BMI were included as covariates in all models. The regression coefficient (β) and two-tailed *p*-value for genotype effects were extracted. To control for multiple testing, Benjamini–Hochberg correction was applied, with false discovery rate (FDR)-adjusted *p*-values (FDR *p* < 0.05) considered statistically significant.

## 3. Results

### 3.1. Genotype Distribution and Hardy–Weinberg Equilibrium Test

The genotype distribution and population genetic equilibrium assessment of GALNT13 rs10196189 in the control group are summarized in Table 2. The observed genotype frequencies were as follows: AA = 125 (89.92%), AG = 13 (9.35%), and GG = 1 (0.71%). The corresponding allele frequencies were A = 94.60% and G = 5.39%. The Hardy–Weinberg equilibrium (HWE) test yielded a result of *p* = 0.329, indicating that the genotype distribution conformed to population genetic equilibrium.

The minor allele frequency (MAF) of the G allele for rs10196189 in the control group was 0.054, which is highly consistent with the reference value of 0.0743 recorded in the PGG.Han v2.0 database for the Han Chinese population. This alignment further supports the population representativeness and genotyping quality of the current study sample, providing a robust basis for subsequent genetic association analyses.

### 3.2. Population- and Sex-Level Differences in Allele Frequencies of rs10196189

Comparative analysis of rs10196189 allele frequencies between the Han Chinese control group and various populations from the gnomAD database revealed significant differences across both ancestral and sex-based groups (Table 3) [29]. Specifically, the frequency of the G allele in the Han Chinese controls was 5.4%, which was significantly lower than in nearly all gnomAD reference populations. The most pronounced differences were observed in comparisons with African-American (χ^2^ = 90.14, *p* = 2.22 × 10^−21^), Amish (χ^2^ = 62.99, *p* = 2.07 × 10^−15^), and Middle Eastern populations (χ^2^ = 47.54, *p* = 5.40 × 10^−12^). Additionally, significant differences were also found when compared to South Asian, Ashkenazi Jewish, and Admixed American populations (all *p* < 0.001). Notably, even when compared to the East Asian group within gnomAD—an intra-continental population—the difference remained statistically significant (χ^2^ = 5.60, *p* = 0.018), suggesting the presence of population-specific allele variation even within broader continental categories.

In contrast, no statistically significant difference was found between the Han Chinese and the European Finnish population (χ^2^ = 0.97, *p* = 0.324), indicating a relatively similar allele distribution between these groups at this locus.

Furthermore, sex-based analysis revealed a significant difference in rs10196189 allele frequencies between genetically female (XX: G = 15,150; A = 62,606) and male (XY: G = 13,810; A = 60,600) individuals in gnomAD (χ^2^ = 21.04, *p* = 4.50 × 10^−6^), suggesting the presence of potential sex-specific variation in the genetic distribution of this polymorphism.

### 3.3. Differences in Genotype and Allele Frequencies Between Athlete and Control Groups

The distribution of GALNT13 rs10196189 genotypes showed a marginal statistical difference between the athlete and control groups (χ^2^ = 4.85, degrees of freedom = 2, *p* = 0.088), and the Fisher’s exact test also approached significance (*p* = 0.069). Further analysis of allele frequencies revealed that the G allele frequency was significantly higher in the athlete group than in the control group (12.2% vs. 5.4%). This difference reached statistical significance in both the Pearson chi-square test (χ^2^ = 4.12, *p* = 0.042) and Fisher’s exact test (*p* = 0.037). The corresponding odds ratio (OR) was 0.41 (95% CI: 0.17–0.999), suggesting a potential enrichment of G allele carriers in the speed–power elite athlete population. These findings indicate that the G allele of GALNT13 rs10196189 may be associated with athletic performance traits. (Table 4).

### 3.4. Group Comparisons of Physical and Physiological Variables Between Athletes and Controls

The athlete group demonstrated significant advantages across a wide range of morphological and functional indicators (Table 5). In terms of body composition, athletes exhibited a significantly lower body fat percentage (15.67% vs. 19.10%, *p* < 0.001, r = 0.387) and fat mass (*p* = 0.0033) compared to controls, while showing higher muscle mass (*p* < 0.001, d = −0.557) and lean body mass (*p* < 0.001, r = −0.342). Bone mineral density was also higher in the athlete group (1.34 vs. 1.29 g/cm^2^, *p* = 0.0122, d = −0.408). Although no significant differences were observed in body weight and BMI (*p* > 0.1), musculoskeletal ultrasound results supported the notion of structural muscular adaptation. Athletes exhibited greater muscle thickness across all major measured muscle groups, especially in the rectus femoris and vastus lateralis (both *p* < 0.001, d > 0.75). Other muscles, including the vastus medialis, semitendinosus, biceps femoris long head, and medial head of the gastrocnemius, also showed significant differences (*p* < 0.05). These structural differences in muscles closely related to lower-limb explosive power may be partially influenced by genetic factors early in development. In terms of functional performance, athletes consistently outperformed controls across critical indicators such as jumping, acceleration, and force output. In jump tests, athletes achieved significantly greater vertical jump heights in all three tasks, reflecting enhanced explosive power and neuromuscular coordination. In the 20 m sprint, athletes completed the sprint in a shorter time (3.04 vs. 3.32 s, *p* < 0.001, r = 0.742)—a large effect size indicating superior innate start speed and acceleration capacity. The isometric mid-thigh pull (IMTP) results further supported this finding: both relative peak force (4.06 vs. 3.32 N/kg, *p* < 0.001) and absolute peak force (*p* < 0.001) were significantly higher in athletes, indicating greater strength reserves and muscle tension output capacity. For maximal strength, 1RM squat values were also markedly higher (153.57 vs. 121.62 kg, *p* < 0.001, r = −0.687), demonstrating outstanding lower-body strength. In the anaerobic power test, the athlete group outperformed controls in both peak and mean power output (both *p* < 0.001), although they also showed a slightly higher fatigue index (*p* = 0.0011). Nevertheless, their high-intensity output potential and training responsiveness remain noteworthy. Overall, long-term systematic training clearly shaped both physical structure and explosive performance. However, whether individuals possess advantageous genetic backgrounds may also influence the degree of adaptation and performance superiority. Therefore, to further investigate whether the rs10196189 polymorphism is associated with athlete status and multidimensional performance traits, we conducted in-depth statistical analyses of this locus.

### 3.5. Logistic Regression Analysis of Genotype and Athlete Status

GALNT13 rs10196189 was found to be significantly associated with athlete status under both the dominant model and the additive allelic model (Table 6). In the dominant model, individuals carrying at least one G allele (i.e., AG or GG genotypes) had a significantly higher likelihood of being classified as athletes compared to AA homozygotes (odds ratio [OR] = 2.58, 95% confidence interval [CI]: 1.04–6.30, *p* = 0.032). Similarly, in the additive allelic model (coded 0/1/2 for number of G alleles), each additional G allele was associated with a higher probability of being an athlete (OR = 2.31, 95% CI: 1.06–5.00, *p* = 0.037). In contrast, the recessive model (GG vs. AA/AG) did not show a significant association (*p* = 0.458).

To further control for potential confounding factors, additional models were constructed, including age and BMI as covariates. The results showed that the GALNT13 rs10196189 polymorphism remained significantly associated with speed–power athlete status under both the dominant and additive models: In the dominant model (AG + GG vs. AA), G allele carriers were 2.53 times more likely to be classified as speed–power athletes than AA homozygotes (OR = 2.53, 95% CI: 1.03–6.10, *p* = 0.039). In the additive model, each additional G allele increased the odds of being an athlete by 2.25 times (OR = 2.25, 95% CI: 1.01–5.03, *p* = 0.045). In comparison, the recessive model (GG vs. AA + AG) still did not reach statistical significance (OR = 2.68, 95% CI: 0.10–68.93, *p* = 0.490). In all models, age and BMI were not significant predictors of athlete status.

### 3.6. Receiver Operating Characteristic (ROC) Curve Analysis

**Figure 2:**
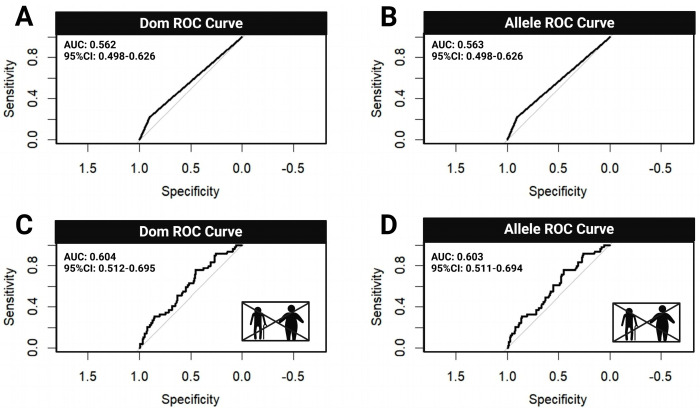
ROC curves of GALNT13 rs10196189 genotype for classifying athlete status. (**A**) ROC curve of the dominant model without covariate adjustment; (**B**) ROC curve of the additive model without covariate adjustment; (**C**) ROC curve of the dominant model adjusted for age and BMI; (**D**) ROC curve of the additive model adjusted for age and BMI. AUC: area under the curve; 95% CI: 95% confidence interval.

To further evaluate the discriminative ability of the genetic models that showed statistically significant associations in the logistic regression analysis, ROC (receiver operating characteristic) curve analyses were performed for the dominant and additive models of GALNT13 rs10196189 (Figure 2). Under the dominant model, the area under the curve (AUC) was 0.562 with a 95% confidence interval of 0.498–0.626, calculated using the DeLong method (Figure 2A). For the additive model, the AUC was 0.563 with the same 95% confidence interval of 0.498–0.626 (Figure 2B). These results indicate a statistically significant association between GALNT13 rs10196189 genotype and athlete status, suggesting that this locus offers valuable predictive information at the individual level. After adjusting for age and BMI as covariates, the dominant model demonstrated an improved AUC of 0.604 (95% CI: 0.512–0.695), indicating moderate classification ability (Figure 2C). Despite modest effect size, the results support the statistical significance of this genotype in predicting athlete status and provide a useful basis for future polygenic score modeling. Likewise, the adjusted additive model yielded an AUC of 0.603 (95% CI: 0.511–0.694), slightly outperforming the dominant model in classification accuracy (Figure 2D). Overall, both models maintained stable predictive performance after adjusting for covariates, highlighting the potential utility of rs10196189 in personalized athlete profiling.

### 3.7. Association Between rs10196189 Genotype and Lower-Limb Performance, Body Composition, and Muscle Morphology

After adjusting for age and BMI, linear regression analysis revealed that the G_carrier genotype of GALNT13 rs10196189 was not significantly associated with the majority of lower-limb strength, power, sprint performance, body composition, or muscle morphology indicators (all FDR-corrected *p* > 0.05). However, medial gastrocnemius thickness showed a nominally significant association with the G_carrier genotype (β = 0.371, *p* = 0.0112; FDR-corrected *p* = 0.1683), suggesting a potential link between this genotype and muscle architecture in specific regions (Table 7). Additionally, some phenotypes, such as IMTP absolute peak force (β = 537.93, *p* = 0.066) and rectus femoris thickness (β = 0.25, *p* = 0.084), demonstrated near-significant trends that may carry biological relevance. Overall, this pilot study did not detect a clear impact of rs10196189 polymorphism on key physical performance and body composition traits in elite male speed–power athletes. Further validation in larger cohorts is warranted.

### 3.8. GALNT13 Tissue Expression Profile and Tissue-Specific Functional Network Analysis

#### 3.8.1. GALNT13 Expression Patterns Across Tissues and Allele-Specific Regulatory Features

**Figure 3:**
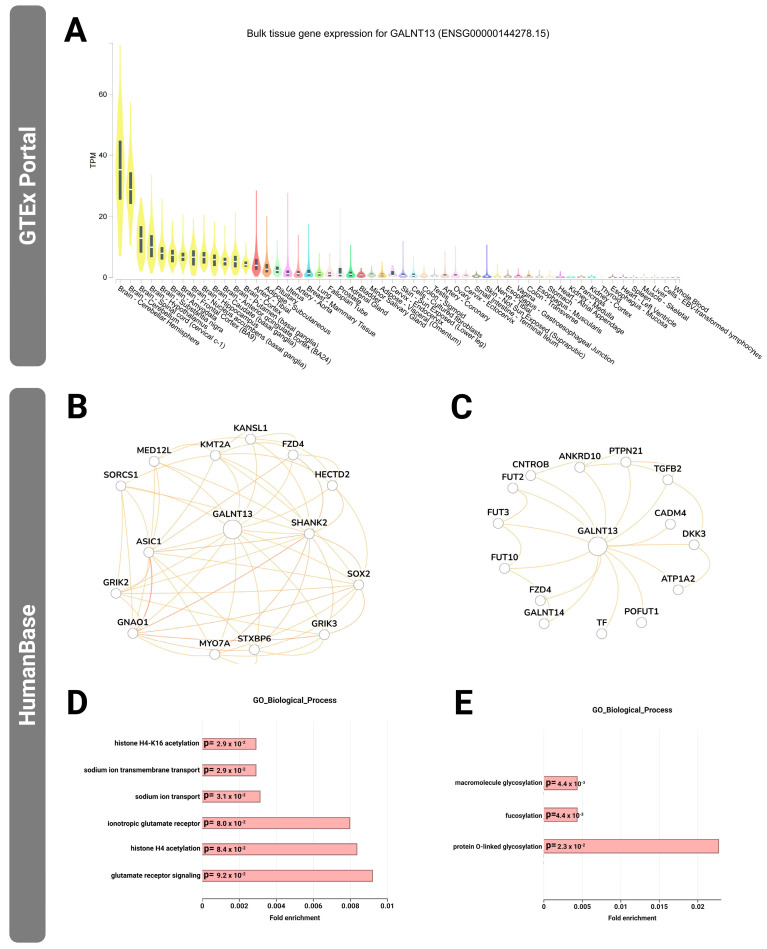
Tissue expression profile, tissue-specific functional interaction networks, and enrichment analysis of the GALNT13 Gene. (**A**) Tissue expression levels of GALNT13 across various human tissues based on the GTEx v8 database (unit: TPM). High expression is predominantly observed in central nervous system-related tissues; (**B**) Tissue-specific functional network of GALNT13 in brain tissue, constructed using the HumanBase platform. Nodes represent functionally related genes, and edges indicate predicted functional interactions; (**C**) Tissue-specific functional network of GALNT13 in skeletal muscle tissue, also constructed using HumanBase; (**D**) GO Biological Process enrichment analysis results based on the gene network shown in Panel B; (**E**) GO Biological Process enrichment analysis results based on the gene network shown in Panel C. GALNT13: Polypeptide N-acetylgalactosaminyltransferase 13; SHANK2: SH3 and multiple ankyrin repeat domains 2; SOX2: SRY-box transcription factor 2; GNAO1: G protein subunit alpha o1; GRIK3: glutamate ionotropic receptor kainate type subunit 3; GRIK2: glutamate ionotropic receptor kainate type subunit 2; STXBP6: syntaxin binding protein 6; MYO7A: myosin VIIA; ASIC1: acid-sensing ion channel subunit 1; SORCS1: sortilin-related VPS10 domain containing receptor 1; KMT2A: lysine methyltransferase 2A; MED12L: mediator complex subunit 12 like; KANSL1: KAT8 regulatory NSL complex subunit 1; FZD4: frizzled class receptor 4; HECTD2: HECT domain E3 ubiquitin protein ligase 2; GALNT14: Polypeptide N-acetylgalactosaminyltransferase 14; FUT3: fucosyltransferase 3; FUT10: fucosyltransferase 10; FUT2: fucosyltransferase 2; TF: transferrin; POFUT1: protein O-fucosyltransferase 1; ATP1A2: ATPase Na^+^/K^+^ transporting subunit alpha 2; DKK3: Dickkopf WNT signaling pathway inhibitor 3; CADM4: cell adhesion molecule 4; TGFβ2: transforming growth factor beta 2; PTPN21: protein tyrosine phosphatase non-receptor type 21; ANKRD10: ankyrin repeat domain 10; CNTROB: centrobin, centrosomal BRCA2 interacting protein.

According to expression analysis based on the GTEx database, GALNT13 is expressed across various human tissues and exhibits pronounced tissue specificity (Figure 3A). Notably, its expression is most prominent in central nervous system-related tissues, including the brain cortex, cerebellar hemisphere, frontal cortex (BA9), and hippocampus, with expression levels generally exceeding 20 TPM (transcripts per million) and reaching above 60 TPM in some brain regions. Outside the brain, GALNT13 also shows moderate expression in a few peripheral tissues, such as the testis and sun-exposed skin (lower leg). In contrast, expression levels in non-neural tissues such as the liver, heart, lung, small intestine, and whole blood are relatively low, approaching background levels, suggesting that the gene’s biological function is likely concentrated in the nervous system. Furthermore, eQTL analysis from GTEx reveals a positive regulatory relationship between the G allele of rs10196189 and GALNT13 expression in the brain cortex (t = 2.2, *p* = 0.027; using the eQTL Calculator at https://gtexportal.org**accessed on 11 July 2025**). This finding supports the hypothesis that the G allele may enhance GALNT13 expression in neural tissues, potentially contributing to the regulation of neurobiological mechanisms underlying athletic performance. However, the tissue expression profile and allele-specific regulatory data provide a strong biological foundation for the subsequent tissue-specific functional network analyses, emphasizing the potential functional relevance of GALNT13 in the nervous system.

#### 3.8.2. Functional Network Construction and Pathway Enrichment Analysis of GALNT13 in Neural and Skeletal Muscle Tissues

To further explore the potential biological functions of GALNT13 across different tissues, we focused on brain and skeletal muscle—two functionally relevant tissue types—based on its tissue-specific expression as revealed by GTEx. We used the HumanBase platform to construct tissue-specific gene interaction networks for GALNT13 (Figure 3B,C) and performed Gene Ontology (GO) Biological Process enrichment analysis (Figure 3D,E).

The selection of these two tissues was based on the following rationale: On one hand, GTEx data (Figure 3A) show significantly elevated expression of GALNT13 in the brain cortex, frontal cortex, and hippocampus, with TPM levels commonly exceeding 20, suggesting its potential role in neurological regulation. On the other hand, previous studies have reported a positive association between GALNT13 expression and the cross-sectional area of fast-twitch fibers in the vastus lateralis muscle [5]. In this study, we also observed a trend-level association between the G allele of rs10196189 and medial gastrocnemius thickness, implying its possible involvement in skeletal muscle structure or adaptive remodeling.

In the neural tissue interaction network constructed via HumanBase (Figure 3B), GALNT13 was tightly connected with genes involved in neurodevelopment and synaptic plasticity, such as SHANK2, SOX2, GRIK3, GNAO1, and STXBP6, suggesting a potential role in neuronal excitability and synaptic stability. In contrast, the skeletal muscle interaction network (Figure 3C) showed GALNT13 functionally co-expressed with glycosyltransferase family members such as FUT3, FUT10, GALNT14, and POFUT1, indicating its involvement in glycosylation-mediated processing of membrane proteins and structural stabilization in muscle tissue.

Further GO Biological Process enrichment revealed tissue-specific enrichment patterns: In brain tissue, GALNT13-associated genes were significantly enriched in pathways related to neuronal signaling and epigenetic modification, including the glutamate receptor signaling pathway (*p* = 6.22 × 10^−6^), ionotropic glutamate receptor signaling (*p* = 6.62 × 10^−6^), sodium ion transport (*p* = 3.14 × 10^−3^), and histone H4-K16 acetylation (*p* = 2.92 × 10^−3^) (Figure 3D). In skeletal muscle, the associated genes were significantly enriched in macromolecule glycosylation (*p* = 4.4 × 10^−3^), fucosylation (*p* = 4.4 × 10^−3^), and protein O-linked glycosylation (*p* = 2.3 × 10^−2^) (Figure 3E), which are closely linked to post-translational modifications. These findings suggest that GALNT13 may exert dual tissue-specific functions: modulating neuronal excitability in the nervous system and glycosylation of membrane proteins in skeletal muscle. This dual role could partially explain its functional relevance in speed–power athletic performance phenotypes.

#### 3.8.3. Integrated Functional Network and Enrichment Analysis

**Figure A1:**
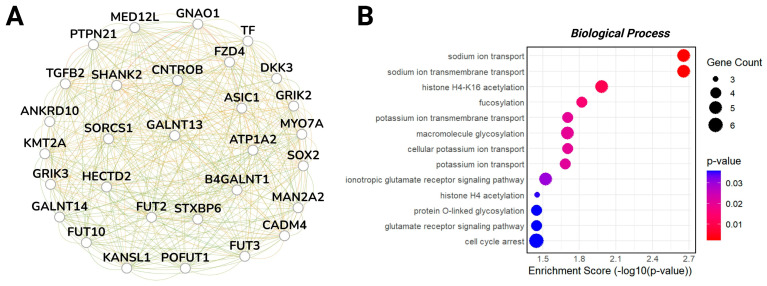
Integrated functional network and enrichment analysis of GALNT13 and its tissue-specific interacting genes; (**A**) Tissue-specific functional interaction networks of GALNT13 in brain and skeletal muscle were constructed using the HumanBase platform, with manual addition of two interacting molecules: B4GALNT1, a glycosyltransferase involved in N-glycan processing and associated with hereditary spastic paraplegia, and MAN2A2, a mannosidase implicated in congenital disorders of glycosylation. Together, these form an integrated network comprising 30 nodes, each representing a gene functionally or physically associated with GALNT13; (**B**) Biological process enrichment analysis based on the 30-gene interaction network. Dot size indicates the number of genes involved in each pathway, and dot color reflects the statistical significance of enrichment, represented as −log10 (*p*-value).

To further explore the potential functional networks of GALNT13 in exercise-related tissues, we constructed its tissue-specific interaction networks in brain and skeletal muscle using the HumanBase platform. Through the GIANT module, we identified the top 15 genes most functionally associated with GALNT13. Additionally, we manually integrated two key glycosylation-related molecules—B4GALNT1 and MAN2A2—based on supporting evidence from network biology: B4GALNT1 was predicted to interact with GALNT13 in the STRING database, while a physical interaction with MAN2A2 was confirmed in BioGRID. Together, these formed a 30-node integrated network encompassing several key biological processes, including neurodevelopment, synaptic transmission, glycosylation, and muscle structure maintenance (Figure A1A).

Based on this network, we conducted a biological process enrichment analysis to identify potential functional modules (Figure A1B). The results revealed significant enrichment of pathways involving sodium/potassium ion transmembrane transport, glutamate receptor signaling pathway, histone H4/H4-K16 acetylation, macromolecule glycosylation, protein O-linked glycosylation, and cell cycle arrest. These pathways play crucial roles in maintaining neuronal excitability, synaptic plasticity, neuromuscular junction development, and energy metabolism regulation, suggesting that GALNT13 may act as a multilayered regulator in shaping individual differences in physical performance by modulating these biological processes.

## 4. Discussion

This pilot study represents the first attempt to replicate the association between the GALNT13 rs10196189 polymorphism and multidimensional athletic-related phenotypes in the world’s largest ethnic group—the Chinese Han population. Notably, all participants in the athlete group were current elite male athletes engaged in speed–power disciplines. Our findings are highly consistent with previous multi-ethnic genome-wide association studies (GWAS), which identified a significant enrichment of the G allele at this locus among elite speed–power athletes [5]. Specifically, the frequency of the G allele was markedly higher in the athlete group than in non-athletic controls (12.2% vs. 5.4%), and both dominant and additive genetic models remained significant predictors of athletic status even after adjusting for age and BMI, highlighting the potential individual-level predictive value of this genotype. Although most associations between genotype and physical performance, body composition, and muscle morphology did not reach statistical significance after FDR correction, a nominally significant association was observed between the G_carrier genotype and medial gastrocnemius thickness (β = 0.371, *p* = 0.0112), suggesting a possible link to specific muscle structural traits.

To further elucidate the population-specific distribution pattern of rs10196189, we systematically compared the G allele frequency observed in the Han Chinese control group (5.4%) with that reported across multiple global reference populations in the gnomAD database (totaling 152,166 alleles) [29]. The results demonstrated that the G allele frequency in the Han population was significantly lower than that in nearly all gnomAD populations, with the most pronounced differences observed in comparisons with African-American (χ^2^ = 90.14, *p* = 2.22 × 10^−21^), Amish (χ^2^ = 62.99, *p* = 2.07 × 10^−15^), and Middle Eastern populations (χ^2^ = 47.54, *p* = 5.40 × 10^−12^) (Table 3). Significant differences were also evident when compared to South Asian, Ashkenazi Jewish, Admixed American, and Non-Finnish European populations (all *p* < 0.001). Notably, even within the broader Asian continental group, a statistically significant difference remained between the Han Chinese and the East Asian subgroup in gnomAD (χ^2^ = 5.60, *p* = 0.018), suggesting the existence of population-specific genetic divergence within continental boundaries. In contrast, no significant difference was observed between the Han Chinese and the European Finnish population (χ^2^ = 0.97, *p* = 0.324), indicating a relatively similar allelic distribution at this locus (Table 3). This low-frequency characteristic of rs10196189 in the Han Chinese, as revealed through a population genetics lens, provides critical background support for our observation of G allele enrichment in elite speed–power athletes. It further underscores the potential of this variant as a valuable genetic marker in exercise genomics research within East Asian populations.

From a biological mechanism perspective, expression data from the GTEx database indicate that the G allele of rs10196189 significantly upregulates GALNT13 expression in the human cerebral cortex (t = 2.2, *p* = 0.027). Moreover, GALNT13 shows pronounced tissue specificity, with especially high expression in central nervous system tissues such as the cerebral cortex, hippocampus, and cerebellar hemisphere (TPM levels > 20), implying a potential role in neuromuscular control. Prior studies of other GALNT family members suggest that this class of polypeptide N-acetylgalactosaminyltransferases may play non-redundant roles in neurodevelopment and motor coordination. For instance, in GALNT2-related congenital disorders of glycosylation (GALNT2-CDG), patients commonly present with phenotypes involving neurodevelopmental delay, autistic traits, hypotonia, and cerebellar dysfunction, while animal models show deficits in motor and sensory integration [30]. These findings highlight the profound impact of glycosylation defects on neuromuscular system development and function, suggesting that GALNT13 may contribute through similar mechanisms.

Furthermore, research in oncology has demonstrated that GALNT family enzymes can regulate EGFR O-linked glycosylation and phosphorylation status, thereby activating the PI3K/Akt/mTOR signaling pathway to modulate cellular proliferation and migration [31]. This pathway also plays a central role in skeletal muscle cell growth, differentiation, and fiber type switching. For example, the corneal proteoglycan Keratocan was found to promote satellite cell proliferation and myogenic differentiation via this pathway, increasing fast-twitch fiber composition and improving muscle mass and function in mice [32]. The functional network of GALNT13 in muscle tissue—particularly its interactions with glycosyltransferases like GALNT14 and POFUT1—supports its potential involvement in similar glycosylation-related mechanisms that regulate membrane protein stability and muscle adaptation.

It is also worth noting that in certain pathological conditions, such as secondary dystroglycanopathies, glycosylation defects result in α-dystroglycan dysfunction and muscle structural degeneration. Research has shown aberrant activation of the mTOR pathway in such contexts, and its inhibition can significantly reduce inflammation, fibrosis, and muscle damage [33]. Collectively, these findings point to a tightly coupled relationship between glycosylation modifications and the Akt/mTOR signaling axis, with dual regulatory effects on skeletal muscle integrity and athletic function in both physiological and pathological states. Based on these mechanistic clues, we hypothesize that GALNT13, as a tissue-specific glycosyltransferase within the neuro–muscular pathway, may exert multidimensional biological effects during the development of explosive athletic performance by regulating synaptic plasticity, membrane protein glycosylation, and the downstream PI3K/Akt/mTOR pathway.

Further insights from the tissue-specific functional network constructed via the HumanBase platform reveal that in brain tissue, GALNT13 forms a tightly connected network with a suite of genes involved in synaptic plasticity, neurodevelopment, and neurotransmitter signaling pathways. These include SHANK2, SOX2, GNAO1, GRIK3, GRIK2, STXBP6, MYO7A, ASIC1, SORCS1, KMT2A, MED12L, KANSL1, FZD4, and HECTD2. Gene Ontology (GO) enrichment analysis of this network shows significant clustering in biological processes such as glutamate receptor signaling, histone modification, and synaptic regulation, suggesting that the GALNT13-centered network is deeply involved in neuronal excitability modulation and motor coordination control. Among these, SHANK2 is a scaffolding protein critical for postsynaptic glutamatergic synapses. Loss of SHANK2 disrupts long-term potentiation (LTP) at parallel fiber–Purkinje cell synapses, leading to impaired motor learning and social interaction deficits [34,35]. SOX2, a master regulator of neural stem cell and glial lineage development, is essential for Purkinje neuron function and motor coordination via its expression in Bergmann glia. Conditional knockout of SOX2 in the cerebellum results in vermis hypoplasia and progressive ataxia phenotypes [36]. GNAO1, encoding one of the most abundant Gα proteins in the central nervous system, has been implicated in a spectrum of neurological disorders ranging from epilepsy to movement disorders. Its function in neurotransmitter release and motor signal transduction is well-established [37]. Importantly, several core genes in the GALNT13 network converge on the glutamate receptor pathway, acting as key intermediaries between genetic variation and excitatory synaptic regulation. For instance, GRIK3 encodes a kainate-type glutamate receptor subunit linked to high neuroticism and is highly expressed in the prefrontal cortex and limbic system. It may indirectly influence athletic performance through its modulation of emotional drive and motor readiness [38]. Similarly, pathogenic mutations in GRIK2 are associated with intellectual disability, dysmyelination, and epilepsy, underscoring its role in excitatory neurotransmission and early motor function development [39]. Other network components represent broader mechanisms of synaptic transmission efficiency, motor behavior regulation, and neuronal energy metabolism. STXBP6 has been identified as a synaptic vesicle release regulator and potential biomarker in Parkinson’s disease dementia, implicating its role in movement execution and memory integration [40]. Recent genetic and functional studies further highlight the critical role of STXBP6 in neurodevelopment. Specifically, de novo mutations in STXBP6 have been linked to developmental epileptic encephalopathy and autism spectrum disorders, reinforcing its classification as a novel SNAREopathy gene [41]. As a brain-enriched SNARE complex component, STXBP6 plays an essential role in synaptic vesicle exocytosis, and loss-of-function mutations may impair neurotransmitter release and neural circuit formation during early development. Additionally, knockout mouse models have revealed that while Stxbp6-null mice survive to adulthood with relatively normal behavior, they display lower body weight and significant alterations in cerebral cortical gene expression profiles, especially in pathways related to immune response and synaptic regulation [42]. Collectively, these findings emphasize the importance of STXBP6 in both synaptic function and neural system homeostasis. Given its potential impact on synaptic transmission and motor coordination, STXBP6 represents a mechanistically plausible link between neuronal development and athletic phenotype variation, warranting further investigation within performance genomics.

MYO7A, an unconventional myosin expressed in auditory and vestibular hair cells, is essential for sensory-motor coupling; mutations result in coordination deficits [43]. ASIC1a, a proton-sensitive ion channel, modulates the CaMKII and ERK pathways in the striatum and is central to procedural learning and motor memory. Knockout models exhibit motor performance impairments [44]. Furthermore, the GALNT13 brain network includes key regulators of neuronal polarity and synaptogenesis. SORCS1 controls the axonal distribution of neurexin, influencing synaptic differentiation and efficiency in motor pathways [45]. KMT2A and MED12L, involved in chromatin remodeling and transcription regulation, respectively, are essential for synaptic development and motor–speech coordination, with dysfunctions linked to developmental delays [46,47]. KANSL1 regulates neuronal autophagy and oxidative stress balance, with animal models showing abnormal motor behavior and mitochondrial damage upon dysfunction [48]. FZD4, a core receptor in the Wnt pathway, is vital for blood–brain barrier integrity and neural stability; its endocytic defects may lead to neuronal damage [49]. Finally, HECTD2, a ubiquitin ligase, is implicated in corpus callosum malformation and hippocampal abnormalities and has been associated with autistic features and attention deficit disorders [50]. Collectively, this GALNT13-centered neuronal network reflects a multi-pathway integration spanning synaptic plasticity, neuronal polarity, glutamatergic transmission, chromatin remodeling, and mitochondrial homeostasis. These mechanisms provide a compelling theoretical and mechanistic basis for GALNT13’s role in brain–muscle coordination and explosive athletic performance. Future studies integrating phenotype–brain imaging data and molecular regulation of GALNT13 could further elucidate its unique role in the fine-tuned regulation of physical performance.

In skeletal muscle tissue, GALNT13 also exhibits significant co-expression with a variety of enzymatic genes—including GALNT14, FUT3, FUT10, FUT2, FZD4, TF, POFUT1, ATP1A2, DKK3, CADM4, TGFβ2, PTPN21, ANKRD10, and CNTROB. These genes are primarily involved in key post-translational modifications, notably O-linked glycosylation and fucosylation, suggesting that this co-expression network may play a role in maintaining muscle structural stability and coordinating signaling functions. Specifically, GALNT14 has been shown to promote cell proliferation and migration by O-glycosylating its substrate PHB2 and activating IGF1R-mediated growth factor signaling, highlighting a core regulatory mechanism of GALNT family members in glycosylation-dependent pathways [51]. Similarly, FUT3 enhances fucosylation of TGFβR-I, thereby activating the TGFβ pathway and inducing epithelial–mesenchymal transition (EMT), a key step in muscle cell remodeling and migration [52]. Members of the FUT family are also implicated in lipid metabolism and cell fate regulation. For example, circFUT10 promotes preadipocyte proliferation while inhibiting differentiation via the ceRNA–metabolic axis involving let-7c/PPARGC1B [53]. FUT2, on the other hand, enhances YAP1 nuclear translocation and stabilizes mSREBP-1, supporting lipogenesis and metabolic reprogramming via fucosylation [54].

Together, these findings support the existence of a “glycosylation–metabolism–structure” axis, potentially coordinated by GALNT13 and FUT family members, which may help maintain skeletal muscle homeostasis under high-intensity exercise and mediate adaptations to training load.

At the signaling level, FZD4, a key receptor in the WNT pathway, forms specific binding interfaces with DVL2 to activate canonical WNT signaling, which is essential for muscle development, regeneration, and neuromuscular coordination [55]. Transferrin (TF) has been identified as an indirect indicator of mTORC1 activity in muscle cells, reflecting metabolic adaptations induced by exercise and potentially serving as a molecular marker for training status [56]. POFUT1 activates the MAPK and PI3K/Akt cascades to promote proliferation, suggesting a role in muscle repair and hypertrophy [57]. ATP1A2, which encodes the Na^+^/K^+^-ATPase α2 isoform, is highly expressed in both CNS and muscle tissue; mutations can cause hypokalemic periodic paralysis and neuromuscular excitability disorders, underscoring its importance in electrophysiological stability [58]. DKK3, a myokine, regulates mitochondrial function and myotube structure via CKAP4 and may serve as a target for muscle atrophy prevention in conditions such as COPD-related sarcopenia or exercise-induced muscle wasting [59].

In terms of structural integration, CADM4 requires palmitoylation at Cys347 for membrane localization; its loss causes myelin abnormalities and disrupts neuro-muscular signaling, also regulating WNT-β-catenin pathways in glial development, implicating roles in motor system maturation [60]. TGFβ2 can promote MSC-to-tendon-like cell differentiation via the Akt–mTORC1–P70S6K pathway, independent of Smad3, suggesting potential roles in muscle–tendon interface remodeling [61]. Although PTPN21 is considered a weak phosphatase, its regulation of ERK activation indicates a role in muscle motility and stress response [62]. ANKRD10, a MYC co-transcriptional activator, regulates cell migration via alternative splicing, potentially contributing to nuclear–cytoplasmic dynamics in muscle cells [63]. Finally, CNTROB (centrobin) is a centrosomal and microtubule-associated protein; its loss impairs cilia formation and spinal development, hinting at roles in motor–skeletal integration [64].

From a network biology perspective, recent protein–protein interaction databases offer new insights into potential pathways relevant to neuromuscular function. According to predictions from the STRING database, GALNT13 may interact with B4GALNT1, a transferase involved in the biosynthesis of gangliosides GM2 and GD2 [25]. Notably, mutations in B4GALNT1 have been confirmed to cause hereditary spastic paraplegia type 26 (HSP26), a neurodegenerative disorder characterized by progressive lower limb weakness and spasticity [24]. This evidence strengthens the hypothesis that GALNT13 may contribute to the structural and functional maintenance of motor pathways via modulation of glycosphingolipid metabolism.

In addition, curated interaction data from the BioGRID database support a physical interaction between GALNT13 and MAN2A2 [26]. MAN2A2 is a key enzyme in the Golgi apparatus responsible for N-glycan trimming and maturation. Recent studies have linked MAN2A2 dysfunction to a range of neurological phenotypes, including autism spectrum disorders, cognitive delay, and congenital disorders of glycosylation (CDG) [27,28]. These conditions are typically characterized by abnormal protein glycosylation, disrupted neurodevelopment, and frequently involve synaptic or axonal dysfunction.

To further elucidate the biological context of the aforementioned molecular interactions, we performed a biological process enrichment analysis based on the 30-node integrative network centered on GALNT13. The analysis revealed several significantly enriched pathways, including sodium/potassium ion transmembrane transport, glutamate receptor signaling, histone H4/H4-K16 acetylation, macromolecule glycosylation, protein O-linked glycosylation, and cell cycle arrest (Figure A1). These pathways are critically involved in maintaining neuronal excitability, synaptic plasticity, neuromuscular junction formation, and cellular energy regulation. Taken together, the integrated network and enrichment results further support the hypothesis that GALNT13 may function as a multifaceted regulatory factor, linking glycosylation dynamics to neuromuscular phenotypic traits and contributing to inter-individual differences in physical performance capacity.

Although this pilot study offers several promising insights into the association between the GALNT13 rs10196189 polymorphism and athletic phenotypes, certain limitations must be acknowledged. Notably, the relatively small sample size may have limited the statistical power to detect genotype–phenotype associations with small effect sizes. Additionally, to minimize physiological confounding factors, the study was deliberately restricted to Han Chinese males, thereby reducing potential variability introduced by sex-specific biological differences. However, this design choice also precludes direct evaluation of potential sex-specific genetic effects at this locus. To preliminarily explore the sex-based distribution of rs10196189, we conducted a comparative analysis using allele count data from the gnomAD database. The results revealed a statistically significant difference in G allele frequency between genetically female (XX: G = 15,150; A = 62,606) and male (XY: G = 13,810; A = 60,600) individuals (χ^2^ = 21.04, *p* = 4.50 × 10^−6^) (Table 3). Moreover, when compared to our Han Chinese male control group (G = 15; A = 263), the G allele frequency in gnomAD male samples was also significantly higher (χ^2^ = 30.95, *p* = 2.65 × 10^−8^), suggesting the presence of complex genetic variation across both population and sex dimensions (Table 3). Future research should therefore aim to recruit larger, multicenter cohorts that include female participants to comprehensively assess the potential modulation of this polymorphism’s effects by sex. In addition, longitudinal study designs and functional validation approaches—such as transcriptomic profiling and CRISPR-based gene editing models—are warranted to further elucidate the tissue-specific roles of GALNT13 and its mechanistic involvement in the regulation of athletic performance.

However, GALNT13 displays dual, tissue-specific functional pathways: In the central nervous system, it is involved in synaptic regulation, electrophysiological signaling, and neuronal development; In skeletal muscle, it may regulate protein glycosylation, metabolic remodeling, and structural integrity. This integrated network is consistent with its high expression in the brain cortex and aligns with the observed nominal association between the rs10196189 G allele and medial gastrocnemius thickness, further supporting the hypothesis that GALNT13 may function as a potential genetic regulator of explosive athletic performance.

## 5. Conclusions

This pilot study is the first to confirm the association between the GALNT13 rs10196189 polymorphism and elite speed–power athlete status in Chinese Han males. The G allele was significantly more frequent in athletes, and both dominant and additive models effectively predicted athlete classification, even after adjusting for age and BMI. Although no performance traits reached FDR-corrected significance, a nominal association with medial gastrocnemius thickness suggests a possible influence on muscle structure. Tissue-specific analyses revealed that GALNT13 is highly expressed in brain regions involved in motor control and is co-expressed with glycosylation- and metabolism-related genes in skeletal muscle. Enrichment in pathways such as synaptic signaling, O-linked glycosylation, and Akt/mTOR signaling supports a dual role in neuromuscular regulation. These findings provide preliminary evidence that GALNT13 may contribute to individual differences in explosive athletic traits. Given its low allele frequency in Han Chinese and enrichment in elite athletes, rs10196189 holds promise as an ethnicity-specific genetic marker. Future research should validate these findings in larger, diverse cohorts and explore underlying mechanisms through functional studies and longitudinal designs.

## Figures and Tables

**Table 1:** Basic anthropometric characteristics of participants in control and athlete groups *.

Group	Sample Size (n )	Height (m)	Weight (kg)	BMI (kg·m^−2^)	Age (y)
Control	139	1.801 ± 0.067	77.319 ± 9.774	23.81 ± 2.35	22.777 ± 3.769
Athletes	49	1.807 ± 0.072	79.138 ± 7.283	24.274 ± 2.026	22.898 ± 4.459

* Values are presented as mean ± standard deviation. Height was reverse-calculated from DXA-derived weight and BMI. BMI = body mass index; DXA = dual-energy X-ray absorptiometry; y = years; m = meters; kg = kilograms.

**Table 2:** Genotype and allele distributions, HWE analysis, and minor allele frequencies for GALNT13 rs10196189 in the control group *.

SNP Locus	Genotype Distribution (n , %)	Allele Distribution (n, %)	HWE *p*	Observed MAF	Reference MAF
GALNT13 rs10196189	AA = 125 (89.92) AG = 13 (9.35) GG = 1 (0.71)	A = 263 (94.60) G = 15 (5.39%)	0.329	0.054 (G)	0.0743 (G)

* HWE *p*-values were calculated using the exact Hardy–Weinberg equilibrium test; *p* > 0.05 indicates consistency with population equilibrium [17]. Observed MAF values were derived from the present control sample. MAF = minor allele frequency; HWE = Hardy–Weinberg equilibrium; SNP = single nucleotide polymorphism.

**Table 3:** Comparative allele frequency analysis of rs10196189 between Han Chinese controls and gnomAD populations with emphasis on ancestral and sex-based differences.

Comparison	G_Han	A_Han	G_Pop	A_Pop	Chi-Square	*p*-Value
Han vs. Total	15	263	28,960	123,206	32.6379	1.11 × 10^−8^
Han vs. African_American			13,373	28,105	90.1368	2.22 × 10^−21^
Han vs. Amish			260	650	62.9973	2.07 × 10^−15^
Han vs. Middle_Eastern			80	214	47.5356	5.40 × 10^−12^
Han vs. South_Asian			1164	3658	50.9074	9.68 × 10^−13^
Han vs. Remaining			413	1697	32.6075	1.13 × 10^−8^
Han vs. Ashkenazi_Jewish			624	2846	27.9504	1.25 × 10^−7^
Han vs. European_NonFinnish			10,059	57,927	18.7074	1.52 × 10^−5^
Han vs. Admixed_American			2046	13,238	14.4862	1.41 × 10^−4^
Han vs. East_Asian			513	4675	5.5986	1.80 × 10^−2^
Han vs. European_Finnish			428	10196	0.9717	3.24 × 10^−1^
Han vs. XY			XY: 13,810	XY: 60,600	30.9504	2.65 × 10^−8^
XX vs. XY (Sex-based)			XX: 15,150; XY: 13,810	XX: 62,606; XY: 60,600	21.0384	4.50 × 10^−6^

XX: Female individuals in gnomAD; XY: Male individuals in gnomAD; Chi-Square: Pearson’s chi-square test statistic; *p*-value: Two-sided *p*-value from chi-square test; Pop: populations.

**Table 4:** Comparison of genotype and allele frequencies of GALNT13 rs10196189 between athlete and control groups.

SNP	Genotype Distribution (Athletes; Controls)	Genotype Test χ^2^ (*p*)	Fisher *p*	Minor Allele Frequency (Athletes; Controls)	Allele Test χ^2^ (*p*)	Fisher *p*	OR (95% CI)
GALNT13 rs10196189	AA = 38, AG = 10, GG = 1; AA = 125, AG = 13, GG = 1	4.85 (*p* = 0.088)	0.069	G: 12.2%; 5.4%	4.12 (*p* = 0.042 *)	0.038 *	OR = 0.41 (0.17–0.999)

* *p* < 0.05 was considered statistically significant. OR = odds ratio; CI = confidence interval; SNP = single nucleotide polymorphism.

**Table 5:** Between-group comparisons of body composition, structure, and physical performance indicators (athletes vs. vontrols) *†.

Variable	Athletes (Mean ± SD)	Controls (Mean ± SD)	Shapiro–Wilk *p* (Athlete/Control)	Test Type	*p *-Value	Effect Size	Effect Type
Squat 1RM (kg)	153.57 ± 19.73	121.62 ± 24.24	0.2845/0.0172	u	<0.0001 *	−0.687	r
IMTP Relative Peak Force (N/kg)	4.06 ± 0.79	3.32 ± 0.53	0.0006/<0.0001	u	<0.0001 *	−0.612	r
IMTP Absolute Peak Force (N)	3142.63 ± 837.66	2550.44 ± 604.52	0.2061/<0.0001	u	<0.0001 *	−0.5	r
CMJ Jump Height (cm)	49.35 ± 5.68	40.85 ± 6.15	0.2442/0.1898	t	<0.0001 *	−1.41	d
SJ Jump Height (cm)	43.98 ± 5.62	38.05 ± 6.96	0.9362/0.7665	t	<0.0001 *	−0.893	d
DJ Jump Height (cm)	38.05 ± 7.52	27.16 ± 8.26	0.6256/0.4785	t	<0.0001 *	−1.349	d
20 m Sprint Time (s)	3.04 ± 0.17	3.32 ± 0.18	0.0028/0.0019	u	<0.0001 *	0.742	r
Peak Anaerobic Power (W)	1050.41 ± 158.99	846.94 ± 189.48	0.8123/0.8155	t	<0.0001 *	−1.117	d
Mean Anaerobic Power (W)	668.04 ± 76.1	579.65 ± 100.67	0.894/0.5819	t	<0.0001 *	−0.931	d
Anaerobic Power Fatigue Index (%)	54.94 ± 11.1	49.64 ± 10.29	0.0015/0.0124	u	0.0011 *	−0.315	r
DXA Weight (kg)	79.14 ± 7.28	77.32 ± 9.77	0.4266/0.023	u	0.1176	−0.151	r
Body Fat Percentage (%)	15.67 ± 4.68	19.1 ± 5.01	0.0606/0.0009	u	0.0001 *	0.387	r
Fat Mass (g)	12107.73 ± 4300.24	14310.37 ± 4897.64	0.0074/<0.0001	u	0.0033 *	0.283	r
Muscle Mass (g)	63064.2 ± 5426.97	59103.28 ± 7616.57	0.1288/0.1748	t	0.0001 *	−0.557	d
Lean Body Mass (g)	65997.96 ± 6384.96	62287.78 ± 7617.4	0.005/0.6673	u	0.0004 *	−0.342	r
BMI (kg/m^2^)	24.27 ± 2.03	23.81 ± 2.35	0.0111/0.0019	u	0.2133	−0.12	r
Bone Mineral Density (g/cm^2^)	1.34 ± 0.11	1.29 ± 0.12	0.6317/0.6451	t	0.0122 *	−0.408	d
Rectus Femoris Thickness (cm)	2.05 ± 0.38	1.79 ± 0.33	0.1505/0.0944	t	0.0001 *	−0.754	d
Vastus Medialis Thickness (cm)	2.78 ± 0.38	2.58 ± 0.33	0.1235/0.0033	u	0.0124 *	−0.24	r
Vastus Lateralis Thickness (cm)	2.51 ± 0.32	2.26 ± 0.31	0.6696/0.4703	t	<0.0001 *	−0.803	d
Semitendinosus Thickness (cm)	2.32 ± 0.47	2.16 ± 0.51	0.4733/0.0028	u	0.0369 *	−0.201	r
Biceps Femoris (Long Head) Thickness (cm)	2.55 ± 0.46	2.4 ± 0.39	0.6714/0.0098	u	0.0494 *	−0.189	r
Medial Gastrocnemius Thickness (cm)	2.14 ± 0.4	2.0 ± 0.32	0.2519/0.7237	t	0.0295 *	−0.41	d

† Values are expressed as mean ± standard deviation (SD). Normality was assessed using the Shapiro–Wilk test. Test type abbreviations: t = independent samples *t*-test; u = Mann–Whitney U test. Effect size abbreviations: d = Cohen’s d; r = rank-biserial correlation. Performance metrics: 1RM = one-repetition maximum; IMTP = isometric mid-thigh pull; CMJ = countermovement jump; SJ = squat jump; DJ = drop jump; W = Wattbike anaerobic power. * *p* < 0.05 is considered statistically significant.

**Table 6:** Association between genotypes and athlete status: Logistic regression results *.

SNP	Genetic Model	OR	95% CI	*p*-Value
GALNT13 rs10196189	Dominant	2.58	1.04–6.30	0.032 *
	Recessive	2.88	0.24–17.02	0.458
	Additive	2.31	1.06–5.00	0.037 *
	Dominant1	2.53	1.03–6.10	0.032 *
	Recessive1	2.68	0.10–68.93	0.490
	Additive1	2.25	1.01–5.03	0.045 *

OR = odds ratio; CI = confidence interval; * *p* < 0.05 is considered statistically significant. OR > 1 indicates increased odds of being classified as an athlete, whereas OR < 1 suggests a potential protective association against athlete; 1 indicates the regression model results after adjusting for age and BMI.

**Table 7:** Association between GALNT13 rs10196189 G_carrier genotype and physical performance, body composition, and muscle morphology traits.

Phenotype	β	*p*	FDR-*p*
Squat 1RM (kg)	−10.3176	0.1106	0.3784
IMTP Relative Peak Force (N/kg)	0.3863	0.1614	0.4062
IMTP Absolute Peak Force (N)	537.9287	0.0657	0.3784
CMJ Jump Height (cm)	−4.0988	0.069	0.3784
SJ Jump Height (cm)	−1.1941	0.5717	0.6988
DJ Jump Height (cm)	0.6365	0.818	0.8708
20 m Sprint Time (s)	0.0578	0.3296	0.5438
Peak Anaerobic Power (W)	−44.4161	0.4339	0.641
Mean Anaerobic Power (W)	−25.0937	0.388	0.6097
Anaerobic Power Fatigue Index (%)	−4.6823	0.2645	0.4848
DXA Weight (kg)	0.4371	0.8478	0.8743
Body Fat Percentage (%)	0.9667	0.4856	0.641
Fat Mass (g)	731.213	0.5296	0.6721
Muscle Mass (g)	−2628.768	0.1848	0.4065
Lean Body Mass (g)	−1713.446	0.467	0.641
Bone Mineral Density (g/cm^2^)	−0.0018	0.9617	0.9617
Rectus Femoris Thickness (cm)	0.25	0.0839	0.3784
Vastus Medialis Thickness (cm)	0.1688	0.25	0.4848
Vastus Lateralis Thickness (cm)	0.186	0.119	0.3784
Semitendinosus Thickness (cm)	−0.0488	0.7869	0.8656
Biceps Femoris (Long Head) Thickness (cm)	0.2457	0.1511	0.4062
Medial Gastrocnemius Thickness (cm)	0.3714	0.0112 *	0.1683

β represents the regression coefficient for the G_carrier genotype (AG/GG vs. AA), adjusted for age and BMI; *p*-values were derived from linear regression models without correction for multiple testing; FDR-*p* represents the *p*-value adjusted for multiple comparisons using the Benjamini–Hochberg procedure. * Indicates significance at *p* < 0.05.

## Data Availability

The individual-level data generated and analyzed during this study are available from the corresponding author upon reasonable request. Due to privacy concerns and institutional ethical requirements, these data are not publicly available. All participants provided written informed consent, and data access is restricted to protect identifiable personal and genetic information. However, the following publicly available datasets and databases were used to support functional annotation and tissue-specific analysis in this study: GTEx Portal (https://www.gtexportal.org/) for cross-tissue expression profiles and eQTL analysis of GALNT13; HumanBase (https://humanbase.io/) for tissue-specific gene interaction networks, functional predictions, and pathway enrichment analyses using the GIANT module; BRENDA Enzyme Database (https://www.brenda-enzymes.org/) for enzymatic classification and biochemical function of GALNT13 (EC 2.4.1.41); Rhea Database (https://www.rhea-db.org/) for reaction-specific biochemical annotation (e.g., RHEA:23956 and RHEA:52424); ChEBI Database (https://www.ebi.ac.uk/chebi/) for chemical entities associated with glycosylation (e.g., UDP-GalNAc: CHEBI:15378; glycopeptides: CHEBI:58223, CHEBI:67138, CHEBI:29999); STRING Database (https://string-db.org/) for predicted protein–protein interactions involving GALNT13 and B4GALNT1; BioGRID (https://thebiogrid.org/) for experimentally validated physical interactions, including those between GALNT13 and MAN2A2; gnomAD (https://gnomad.broadinstitute.org/) for global allele frequency comparisons across populations and sexes; PGG.Han v2.0 (https://www.biosino.org/pgghan2) for reference allele frequencies in the Han Chinese population. These resources were integrated to support the interpretation of genetic association results and to explore the tissue-specific functional context of GALNT13.

## Abbreviations

The following abbreviations are used in this manuscript:

1RM	One repetition maximum
Akt	Protein kinase B
ANKRD10	Ankyrin repeat domain 10
ASIC1	Acid-sensing ion channel subunit 1
ATP1A2	ATPase Na^+^/K^+^ transporting subunit alpha 2
AUC	Area under the curve
B4GALNT1	Beta-1,4-N-acetylgalactosaminyltransferase 1
BioGRID	Biological General Repository for Interaction Datasets
BMD	Bone mineral density
BMI	Body mass index
CADM4	Cell adhesion molecule 4
CDG	Congenital Disorders of Glycosylation
ceRNA	Competing endogenous RNA
ChEBI	Chemical Entities of Biological Interest
CI	Confidence interval
CMJ	Countermovement jump
CNTROB	Centrobin, centrosomal BRCA2 interacting protein
CNV	Copy number variation
CRISPR	Clustered Regularly Interspaced Short Palindromic Repeats
DJ	Drop jump
DKK3	Dickkopf WNT signaling pathway inhibitor 3
DNA	Deoxyribonucleic acid
DNA-seq	DNA sequencing
DXA	Dual-energy X-ray absorptiometry
EC	Enzyme commission number
EMT	Epithelial–mesenchymal transition
eQTL	expression quantitative trait locus
FDR	False discovery rate
FUT10	Fucosyltransferase 10
FUT2	Fucosyltransferase 2
FUT3	Fucosyltransferase 3
FZD4	Frizzled class receptor 4
GALNT13	Polypeptide N-acetylgalactosaminyltransferase 13
GALNT14	Polypeptide N-acetylgalactosaminyltransferase 14
GNAO1	G protein subunit alpha o1
gnomAD	Genome Aggregation Database
GO	Gene Ontology
GRIK2	Glutamate ionotropic receptor kainate type subunit 2
GRIK3	Glutamate ionotropic receptor kainate type subunit 3
GTEx	Genotype-Tissue Expression
GWAS	Genome-wide association study
HECTD2	HECT domain E3 ubiquitin protein ligase 2
HWE	Hardy–Weinberg equilibrium
IMTP	Isometric mid-thigh pull
KANSL1	KAT8 regulatory NSL complex subunit 1
kg	Kilograms
KMT2A	Lysine methyltransferase 2A
LTP	Long-term potentiation
m	Meters
MAF	Minor allele frequency
MAN2A2	Alpha-Mannosidase II
MED12L	Mediator complex subunit 12 like
mTOR	Mechanistic target of rapamycin
MYO7A	Myosin VIIA
NSCA-CPSS	National Strength and Conditioning Association-Certified Personal Sports Specialist
NSCA-CSCS	National Strength and Conditioning Association-Certified Strength and Conditioning Specialist
OR	Odds ratio
PI3K	Phosphoinositide 3-kinase
POFUT1	Protein O-fucosyltransferase 1
PTPN21	Protein tyrosine phosphatase non-receptor type 21
Rhea	Rhea: Expert-curated biochemical reactions database
RNA	Ribonucleic acid
ROC	Receiver operating characteristic
SD	Standard deviation
SHANK2	SH3 and multiple ankyrin repeat domains 2
SJ	Squat jump
SNARE	Soluble NSF attachment protein receptor
SNPs	Single nucleotide polymorphisms
SORCS1	Sortilin-related VPS10 domain containing receptor 1
SOX2	SRY-box transcription factor 2
SREBP-1	Sterol Regulatory Element Binding Protein 1
STRING	Search Tool for the Retrieval of Interacting Genes/Proteins
STXBP6	Syntaxin binding protein 6
TF	Transferrin
TGFβ2	Transforming growth factor beta 2
TPM	Transcripts per million
y	Years
